# Supplementary material for: Importance of Basic Research on the Causes of Feather Pecking in Relation to Welfare
Source: Animals (Basel). 2020 Jan 28;10(2):213. doi: 10.3390/ani10020213 (PMC7070775; doi:10.3390/ani10020213)
Supplement: Supplementary file 1 [file animals-10-00213-s001.pdf]

animals

MDPI

Supplementary material

Table S1 | A non-systematic overview of publications on topics relating feather pecking in laying hens. Expansion on Table 1.

Abbreviations: serotonin (5-HT); somatodendritic 5-HT 1A autoreceptor agonist (S-15535); Aggressive pecking (AP); beak trimming (BT); body weight (BW); corticosterone (CORT); Dopamine (DA); Emerge box (EB); environmental enrichment (EE); extreme feather pecking (EFP); feather pecking (FP); gentle feather pecking (GFP); high feather pecking (HFP); heterophil/lymphocyte (H/L) ratio; heart rate variability (HRV); human serum albumin (HuSA); low feather pecking (LFP); lipopolysaccharide (LPS); novel object (NO); open field (OF); plumage damage (PD) parent-stock (PS); Tonic immobility (TI); L-tryptophan (TRP).

Strain(-type)s: Columbian Blacktail (CB); Dekalb White (DW); ISA brown (ISA); Lohmann Brown (LB); Lohmann Brown-Classic (LBC); Lohmann Selected Leghorn (LSL); Norbrid 41 (NB); New Hampshire (NH); Rhode Island Red (RIR); Rhode Island White (RIW); White Leghorn (WL); Warren SSL (WSSL).

Reference	Experiment	Subjects	Measured	Results / Conclusion(s)
**Environment**				
**Blokhuis & van der Haar, 1989 [1]**	2*2 Factorial design: litter / wire floors * BT / non-BT in rearing period	*Gallus gallus domesticus* (WSSL)	▪ Behavior<br>▪ Plumage quality	▪ Effect of experiences during rearing on pecking preference in the laying period<br>▪ No effect of BT on pecking preference or frequency of FP
**El-Lethey et al., 2000 [2]**	▪ 2*2 Factorial design: foraging material (long-cut straw (y/n)) * food (pellets / mash)<br>▪ Physical restraint test	*Gallus gallus domesticus* (LSL)	▪ Behavior<br>- TI<br>▪ BW<br>▪ Egg production<br>▪ H/L ratio<br>▪ Antibody titers to sheep red blood cells (SRBC), tetanus	▪ Foraging material and food type affected both FP and stress indicators, suggesting an association<br>- Negative effect of foraging material on FP<br>- More FP in groups fed on pellets than on mash<br>- Positive effect of foraging material on egg production<br>- No effect of food type on egg production<br>- Negative effect of foraging material on TI and H/L ratios<br>- Higher TI and H/L ratios in groups fed on pellets than on mash

Reference	Experiment	Subjects	Measured	Results / Conclusion(s)
			toxoid (TT) and human serum albumin (HSA)	- Positive effect of foraging material on antibody titers to SRBC and TT<br>- No effect of food type on antibody titers
**Riedstra & Groothuis, 2004 [3]**	2*2 Factorial design: light / dark during incubation * housed with familiar conspecifics / housed with both familiar and unfamiliar conspecifics	*Gallus gallus domesticus*	▪ Behavior<br>- Frequency of FP<br>- Social orientation of FP	▪ Light-exposure during incubation: more FP, no FP preference<br>▪ No light-exposure during incubation: less FP, FP preference for unfamiliar over familiar peers
**van Hierden et al., 2004[4]**	2*2 Factorial design: line (LFP / HFP) * diet (low TRP (control) /high TRP) Physical restraint test	*Gallus gallus domesticus* (WL; HFP, LFP)	▪ Behavior<br>▪ plasma-CORT<br>▪ TRP and other large amino acids (LNAAs)<br>▪ 5-HT	▪ No significant interactions effects of line * treatment<br>▪ Negative effect of TRP on frequency GFP (sign.) and SFP (not sign.)<br>▪ HFP higher levels of GFP and SFP than LFP<br>▪ Positive effect of TRP on plasma-TRP/LNAA ratio<br>▪ Positive effect of TRP on baseline and stress-induced plasma-CORT<br>▪ Positive effect of TRP on 5-HT turnover in the forebrain<br>▪ FP is triggered by low serotonergic neurotransmission
**Chow & Hogan, 2005 [5]**	Repeated experience with exploratory-rich environments vs. no such experience	*Gallus gallus spadiceus*	▪ Behavior<br>- Frequency of FP	▪ Effect of experience in the exploratory-rich environments: less GFP, more SFP<br>▪ No effect on frequency of environmental pecking or food pecking

**Reference**	**Experiment**	**Subjects**	**Measured**	**Results / Conclusion(s)**
**McAdie & Keeling 2005 [6]**	1) Experimental condition: device presentation in pen (continuously from 1 day of age / 4 h per day from 1 day of age / from 22 days of age / from 52 days of age / devices never presented)<br>2) Commercial condition: device presentation in pen (continuously from 1 day of age / 24 h per day every 4 weeks / continuously from 16 weeks of age / devices never presented)	*1) Gallus gallus domesticus* (WL; HFP)<br>*2) Gallus gallus domesticus* (LSL)	1) Behavior<br>2) Plumage quality	▪ 'String device an effective enrichment strategy for reducing FP:<br>1) Negative effect on FP when devices continuously in pen from 1 day of age or when they were presented for 4 h per day<br>1) Highest FP in pens where device was never presented, intermediate when introduction was at 22 or 52 days of age<br>2) Negative effect on PD, all ages
**Zimmerman et al., 2006 [7]**	▪ Six treatments with variations in stocking density (low / medium / high) * flock size (small / large) * management type (standard / modified: nipple line drinkers, dark nest boxes)<br>▪ Each pen contained one replicate of a treatment	*Gallus gallus domesticus* (Shavers)	▪ Behavior	▪ No effect of stocking density on welfare<br>▪ Low stocking density: highest initial level of FP and aggression<br>▪ High stocking density: FP increased with age<br>▪ High stocking density: more aggression, preening and allopreening in small flocks than in large flocks<br>▪ High stocking density / small flocks / standard management: highest FP and aggression at the end of the cycle than<br>▪ High stocking density / small flocks / modified management: decreased FP and aggression

Reference	Experiment	Subjects	Measured	Results / Conclusion(s)
**Dixon et al., 2008 [8]**	▪ Observe and quantify motor patterns of GFP and SFP (flat / chicken-shaped feather model)<br>▪ Observe and quantify motor patterns of dustbathing ((peat moss / white sand / grey sand) / water) and foraging pecks (flat / water) and foraging pecks (flat / chicken-shaped forage)<br>▪ NO test (flat / chicken-shaped NO)	*Gallus gallus domesticus* (ISA (WL))	▪ Behavior<br>▪ Duration of head fixation<br>▪ Duration from fixation to contact<br>▪ Duration of the whole peck	▪ Significant variance in peck motor patterns at forages, dust baths, NO and water<br>▪ Peck motor patterns different for all measures for dustbathing and foraging<br>▪ SFP similar to foraging pecks, but unlike all other pecks<br>▪ Thus, SFP derives from frustrated motivations to forage, not to dustbathe
**Lambton et al., 2010 [9]**	Observation of free range / organic / barn systems, BT and non-BT	*Gallus gallus domesticus* (CB)	▪ Behavior (GFP/ SFP)<br>▪ Plumage quality<br>▪ Farmer interviews (y/n FP)<br>▪ Environmental and management information<br>- Weather<br>- Inside / outside temperatures<br>- Light levels	▪ GFP rates decreased with increased percentage range use<br>▪ GFP rates decreased with temperature inside the laying house<br>▪ GFP was lower in flocks with straw litter, even compared to saw dust<br>▪ GFP was higher in flocks with soil or grass litter<br>▪ GFP was higher in flocks which had no perch access<br>▪ GFP was higher in flocks which were BT<br>▪ SFP decreased with range use<br>▪ SFP was higher in non-BT<br>▪ SFP was higher in flocks that were observed to be FP when they arrived on farm compared to flocks that were observed not to FP at arrival

Reference	Experiment	Subjects	Measured	Results / Conclusion(s)
			- Litter type, weight, condensity<br>- Range quality<br>- Range coverage<br>- Range usage	▪ SFP was higher in flocks fed pelleted compared to those fed mashed food<br>▪ PD was lower in BT compared to non-BT flocks<br>▪ PD was lower in flocks which were fed mashed feed, and showed a quadratic relationship with SFP which was positive over the observed ranges of the behaviors
**Collins et al., 2011 [10]**	Observation of hen location on wire floor / shavings / perches, peat, nest box and shavings.	*Gallus gallus domesticus* (Hyline)	▪ Behavior<br>▪ Positional data to calculate feeding synchrony and cluster scores	▪ No effect of pen environment on feeding synchrony<br>▪ Resource-use stronger effect on clustering than social cohesion
**Gilani et al., 2012 [11]**	▪ Dark brooders vs. light (control) during rearing<br>▪ NO test<br>▪ Stationary person test	*Gallus gallus domesticus* (CB)	▪ Behavior<br>▪ Plumage quality<br>▪ Bird weights<br>▪ Evenness of body weight<br>▪ Mortality	▪ No detrimental effects of dark brooding:<br>- No effect on weight evenness<br>- Possible reduced smothering during rear<br>- No effect on GFP<br>- Decreased SFP<br>- Decreased PD<br>▪ Small scale results were similar to those of large-scale studies on commercial farms
**Hartcher et al., 2015 [12]**	2*2 Factorial design: EE / no EE * BT / non-BT	*Gallus gallus domesticus* (ISA)	▪ Behavior<br>▪ Plumage quality<br>▪ Feed intake	▪ Negative effect of BT on PD<br>▪ No effect of EE on PD<br>▪ Negative effect of BT on GFP and SFP at 43wk

**Reference**	**Experiment**	**Subjects**	**Measured**	**Results / Conclusion(s)**
			▪ Live body weight<br>▪ Egg production	▪ Positive effect of BT on GFP during rear and subsequently on PD
**Zepp et al., 2018 [13]**	3*2 Factorial design: Varying stocking densities * Varying EE (pecking stone / pecking block / lucerne bale)	*Gallus gallus domesticus* (LBC)	▪ Behavior<br>▪ Plumage quality<br>▪ Age	▪ Positive effect of stocking density on FP<br>▪ Negative effect of EE on FP and AP<br>▪ Plumage quality is a valid indicator of SFP<br>▪ Less FP perches vs. wire or litter floor
**Genotype**				
**Kjear, 2000 [14]**	Observation of behavior and integument condition for two full laying cycles in four hybrid strains (two WL type, two medium heavy type).	*Gallus gallus domesticus* (LSL and NB (WL), LB and ISA)	▪ Behavior<br>▪ Plumage quality<br>▪ Skin quality<br>▪ Keel bone<br>▪ Bumble foot<br>▪ BW<br>▪ Beak length and curve	▪ Medium heavy strains more FP than WL strains<br>▪ ISA more FP than LB<br>▪ No effect of strain on overall bout size or pecks per bout<br>▪ Tail directed bout size longer than for other body parts<br>▪ Dorsal directed bout size longer for WL than for other strains<br>▪ Ventral directed bout size higher for LSL than for other strains
**Kjaer & Sorensen, 2002 [15]**	▪ Genotype * (high vs. low) level of dietary methionine + cystine<br>▪ 3 Genotypes, 2 levels of light intensity, 2 ages of access to the range area	*Gallus gallus domesticus* (ISA, NH, WL and a cross between NH and WL)	▪ Behavior<br>▪ Plumage quality<br>▪ Age<br>▪ Health status of foot and comb<br>▪ Mortality<br>▪ Egg production	▪ Minor effect on pecking behavior:<br>- Dietary level of methionine + cystine<br>- Light intensity during rearing<br>- Age at access to the range area<br>▪ Substantial variation in FP between batches<br>▪ Correlation high FP and high mortality from cannibalism

**Reference**	**Experiment**	**Subjects**	**Measured**	**Results / Conclusion(s)**
			▪ Floor eggs<br>▪ Egg weight<br>▪ Shell quality<br>▪ Temperature<br>▪ Humidity	
**Rodenburg et al., 2003 [16]**	▪ OF test<br>▪ Social test	*Gallus gallus domesticus*	▪ Behavior<br>▪ Body weight<br>▪ Heritability (h^2, estimate sire and dam variances) of behavioral traits	GFP and OF behaviors found heritable, may be used in selection against FP:<br>- h^2 were higher at 5 wks. compared to 29 wks. for OF behaviors<br>- h^2 were higher at 30 wks. compared to 6 wks. for GFP, ground pecking and BW in social test<br>- h^2 estimate for SFP was not significantly different from zero at either age
**Bolhuis et al., 2009 [17]**	▪ 2*2 Factorial design: genetic line (group-selected against mortality or control) * BT / non-BT<br>▪ Physical restraint test<br>▪ Sudden human approach test	*Gallus gallus domesticus* (WL; low mortality and control line)	▪ Behavior<br>▪ Plumage quality<br>▪ Plasma-CORT<br>▪ 5-HT<br>▪ Platelet 5-HT uptake	- Low mortality line showed less fear-related behavior than control<br>- Low mortality line showed higher 5-HT and lower platelet 5-HT uptake: possible differences in functional activity of the 5-HT system<br>- Negative effect of BT on fear and PD<br>- No effect of BT on peripheral 5-HT: fearfulness and 5-HT activity possibly related to FP, without distinguishing between cause and effect.<br>▪ Peripheral 5-HT activity may reflect the predisposition for SFP

Reference	Experiment	Subjects	Measured	Results / Conclusion(s)
**Kjaer & Jorgensen, 2011 [18]**	▪ Physical restraint test<br>▪ Social test	*Gallus gallus domesticus* (WL; HFP, LFP and control)	▪ HRV (by ECG)<br>▪ Sympathovagal balance (by using pharmacological blockades to selectively inhibit the regulatory influences of the different branches of the ANS on cardiac activity)	▪ Significant ANS response induced by both physical restraint and social test<br>▪ Effect physical restraint strongest on HFP line, than on control resp. LFP line (i.e. selection for FP increased the ANS response to physical restraint)<br>▪ Effect social test stronger on HFP line and control, than on LFP line (i.e. selection against FP reduced the ANS reaction to increased social contact)<br>▪ Physical restraint higher stress reaction than social test
**de Haas et al., 2014 [19]**	• Relate behavior and physiological parameters (PD, plasma-CORT and 5-HT) of PS to high levels of SFP and anxiety in offspring<br>• Offspring: housing system (open, partly open, closed) * litter conditions (limitation of litter (yes/no) * disruption of litter supply (yes/no))	*Gallus gallus domesticus* (DW (WL) and ISA (RIR and RIW))	▪ Behavior<br>▪ Plumage quality<br>▪ Basal plasma-CORT<br>▪ 5-HT levels	▪ Effect of PS strain on anxiety and SFP in offspring (highest for DW hybrid):<br>▪ DW: Positive correlation between PS (CORT, PD, 5-HT) and offspring anxiety and SFP<br>▪ Positive effect of disruption and limitation of litter supply during rearing on anxiety and SFP in offspring (highest for ISA hybrid)

**Reference**	**Experiment**	**Subjects**	**Measured**	**Results / Conclusion(s)**
	• NO test<br>• Stationary person test<br>• Social isolation test			
**van der Eijk et al., 2019 [20]**	▪ Measures of innate and adaptive immune characteristics in HFP and LFP lines<br>▪ Test whether differences in immune characteristics were reflected in the relative abundance of immune cell subsets	*Gallus gallus domesticus* (WL; HFP and LFP)	▪ Behavior<br>▪ Nitric oxide production by blood derived monocytes<br>▪ SpAb titers to HuSA<br>▪ IgM and IgG N(A)Ab titers<br>▪ Immune cell subsets	▪ Divergent selection on FP affects different arms of the immune system:<br>- Higher nitric oxide production, higher IgM and IgG specific antibody titers and higher IgG natural (auto)antibody titers) in HFP than in LFP line<br>▪ No effect of divergent selection on FP on the relative abundance of immune cell subsets
**Piepho et al., 2017 [21]**	▪ Analyze data on SFP of seven lines of HFP and LFP and their F_2-cross.<br>▪ Fit a two-component mixture of Poisson distributions to uncover hidden sub-groups of EFP birds.	*Gallus gallus domesticus* (WL; HFP and LFP)	▪ Behavior<br>- Bouts per bird (bpb)	▪ Line effect on mean bpb<br>▪ Proportion of EFP in LFP marginal compared to HFP and F_2-cross<br>▪ EFP also present in LFP<br>▪ SFP in layer flocks is not a homogenous behavior
**Iffland et al., 2019 [22]**	▪ Fit of mixture of two negative binomial distributions to FP data of a F_2-cross	*Gallus gallus domesticus* (WL)	▪ Behavior<br>▪ Latency (EB and TI tests)	▪ Subgroup of EFP made up about one third of the animals<br>▪ EFP birds higher FP frequency and higher FP intensities than non-EFP

**Reference**	**Experiment**	**Subjects**	**Measured**	**Results / Conclusion(s)**
	▪ Subsequent calculation of posterior probability for new trait (pEFP)<br>▪ TI and EB tests at juvenile and adult age		▪ Heritability (estimate) of behavioral traits	▪ pEFP has a heritability of 0.35<br>▪ pEFP is positively correlated with the fear traits
**Phenotype**				
**Albentosa et al., 2003 [23]**	▪ NO test<br>▪ Subsequent treatment: allocation of birds (pen type) by bird type (categorized by mean distance from the NO)<br>▪ Feather bundle tests (loose feather test, fixed feather test)	*Gallus gallus domesticus* (ISA)	▪ Behavior<br>- FP<br>- AP<br>▪ Plumage quality<br>▪ Mean distance from the NO	▪ No correlation between response to NO and FP<br>▪ In pen type 'varied range of responses to NO' more birds performed FP, FP more consistent and more environmental pecking than in pen type 'similar responses to NO'
**de Haas et al., 2013 [24]**	▪ NO test<br>▪ Stationary person test	*Gallus gallus domesticus* (DW (WL) and ISA (RIR and RIW))	▪ Behavior<br>▪ Plumage quality<br>▪ Basal plasma-CORT<br>▪ whole-blood 5-HT levels<br>▪ Group size<br>▪ Production parameters<br>- Laying percentage	▪ DW more fearful of stationary person than ISA<br>▪ DW more PD than ISA<br>▪ DW lower 5-HT levels than ISA<br>▪ Genotypes did not differ in CORT<br>▪ High CORT associated with low egg weight<br>▪ ISA: large group size associated with low feed intake and better feed conversion<br>▪ ISA: high fear of the stationary person associated with high mortality<br>▪ DW: high fear of the NO associated with low body weight, low egg weight, and low feed intake

**Reference**	**Experiment**	**Subjects**	**Measured**	**Results / Conclusion(s)**
			- Egg weight<br>- Feed intake<br>- Feed conversion<br>- Hen body weight<br>- Mortality<br>- Occurrences of smothering events	
**Physiology**				
**van Hierden et al., 2004 [25]**	▪ Different doses of S-15535 * HFP / LFP<br>▪ Physical restraint test	*Gallus gallus domesticus* (WL; HFP and LFP)	▪ Behavior<br>▪ 5-HT and 5-HT metabolite levels<br>▪ DA and DA metabolites levels	▪ S-15535 useful tool for reducing 5-HT turnover in the forebrain of LFP and HFP chicks<br>▪ HFP and LFP similar 5-HT turnover levels (suggesting a comparable number or sensitivity of presynaptic 5-HT 1A autoreceptors)<br>▪ 4.0 mg/kg S-15535 was the most effective dose (without affecting DA turnover)<br>▪ HFP showed higher proactivity during the physical constraint test<br>▪ Acute S-15535 injection increased FP in HFP birds<br>▪ Thus, low serotonergic neurotransmission modulates the performance of FP

**Reference**	**Experiment**	**Subjects**	**Measured**	**Results / Conclusion(s)**
**Parmentier et al., 2009 [26]**	Intratracheal (i.t.) immunization by concurrent intratracheal primary (at 7 wk of age) and secondary (at 13 wk) challenges of layers with (protein antigen HuSA (1 / 0.5 / 0.01 mg / control) and pathogen-associated molecular pattern LPS (0.1 / 0.5 / 0.01 mg / control)), followed (at 11mo) by challenge of 0.01 mg HuSA + 0.5 mg of LPS.	*Gallus gallus domesticus* (LSL (WL))	▪ Plumage quality<br>▪ Body condiition<br>▪ Egg production<br>▪ Humoral Immune Response to HuSA and LPS<br>▪ Total antibody (Ab) titers to HuSA and LPS in plasma	▪ I.t. immunization with a high dosage of HuSA (for all doses of LPS) more PD and less wounds in vent region than birds not receiving HuSA.<br>▪ I.t. immunization with a high dosage of LPS correlated to comb damage<br>▪ Thus, stimulation of specific (humoral) immune responses (to HuSA) rather than innate responses (to LPS) at a young age may predispose layers for FP at later ages.<br>▪ Involvement of immune mechanisms in FP or vent damage may differ
**Kops et al., 2013 [27]**	Measure of brain monoamine levels in four brain areas (medial striatum, hippocampus, dorsal thalamus and arcopallium) for phenotypes (SPFs, victims, non-FPs).<br>Physical restraint test	*Gallus gallus domesticus* (WL; low mortality and control line)	▪ Behavior<br>▪ 5-HT and 5-HT metabolite levels<br>▪ DA and DA metabolites levels	▪ Serotonergic neurotransmission in the dorsal thalamus and striatum depends on FP phenotype:<br>- SFPs and victims higher 5-HT in dorsal thalamus than non-FPs<br>- Non-FPs highest 5-HT in the medial striatum, then SPFs and victims resp.<br>- No effect of phenotype on 5-HT in arcopallium or hippocampus<br>- No effect of phenotype on DA in any of the four brain areas
**Behavior**				

**Reference**	**Experiment**	**Subjects**	**Measured**	**Results / Conclusion(s)**
**Van Hierden et al., 2002 [28]**	Observe and compare behavior of chicks from HFP and LFP lines during the first 8 weeks of life.	*Gallus gallus domesticus* (WL; HFP and LFP)	Behavior	▪ Line differences in FP behavior can be observed from a very early age during development<br>▪ Line effects targeting of pecking behavior<br>- HFP chicks showed more GFP than LFP<br>- HFP showed more preening than LFP<br>- LFP showed longer duration foraging and feeding behaviors than HFP<br>- HFP showed negative correlation between GFP and preening<br>- LFP showed negative correlation between GFP and duration of feeding<br>- Principal component analysis:<br>- HFP showed high and opposite loadings on the same component for GFP and preening<br>- HFP showed loadings on the other component for GFP and feeding<br>- LFP showed opposite loadings on the same component for GFP and feeding<br>- LFP showed the same loadings for GFP and preening
**Cloutier et al., 2002 [29]**	▪ Used of inanimate chicken model as cannibalism stimulus<br>▪ Demonstrators were trained to pierce a membrane covering a	*Gallus gallus domesticus* (WL)	▪ Behavior<br>- Peck latency<br>- Pierce latency<br>▪ Amount of blood disappearing	▪ Social learning can contribute to the spread of cannibalistic behavior in domestic fowl<br>▪ Observing demonstrator piercing membrane and consuming blood increased likelihood of performing this task

**Reference**	**Experiment**	**Subjects**	**Measured**	**Results / Conclusion(s)**
	dish of chicken blood and consume the blood<br>▪ Two observer treatments: observe stimulus through a wire mesh partition / observe stimulus within the same enclosure		from the model during the test<br>▪ Chicken model membrane damage	▪ Direct access to the cannibalism stimulus enhanced learning of the task where observing it through a wire mesh partition did not<br>▪ Direct access to the cannibalism stimulus during demonstrations enhanced blood consumption during tests<br>▪ Observing demonstrator performing task and direct access to the cannibalism stimulus during demonstrations resulted in bigger holes made in the membrane during tests<br>▪ Individual learning occurred in the absence of social learning
**Forkman et al., 2004 [30]**	▪ SFP performing birds / non-FP birds, HFP /LFP lines<br>▪ Owner-intruder test<br>▪ NO test	Not specified	▪ Behavior	▪ FP did not predict agonistic behavior in owner-intruder test<br>▪ non-FP showed less fear and distance to 'intruder'<br>▪ FP did not predict latency to approach the NO test<br>▪ non-FP showed more rapid habituation during NO test<br>▪ HFP more aggressive more aggressive hopping, pecking and kicking than LFP<br>▪ HFP came closer to the NO<br>▪ Line did not predict habituation during NO test
**Harlander-Matauschek et al., 2007 [31]**	Observe preference for mash / wood shavings / downy feathers / empty bowl for: Management system (cage /	*Gallus gallus domesticus* (WL; HFP and LFP)	▪ Behavior<br>- Amount of substrate eaten	▪ Hens were motivated to eat feathers and wood shavings<br>▪ HFP birds have a stronger preference for feathers than LFP birds

Reference	Experiment	Subjects	Measured	Results / Conclusion(s)
	litter) * Line (HFP / LFP) * State (food-deprived / non-food deprived)		- Total duration of pecking into the bowls, manipulating and eating the substrates<br>- Latency to first movement to peck into or eat from the different substrates<br>- The number of visits to the different substrates	- No effect of line on preference for wood shavings<br>- Caged ate more shavings and visited shaivngs more than floor pen<br>- Food deprived HFP and non-food deprived caged HFP ate more feathers than LFP

References

1. Blokhuis, H.J.; Van Der Haar, J.W. Effects of floor type during rearing and of beak trimming on ground pecking and feather pecking in laying hens. *Appl. Anim. Behav. Sci.* **1989**, *22*, 359–369.
2. El-Lethey, H.; Aerni, V.; Jungi, T.W.; Wechsler, B. Stress and feather pecking in laying hens in relation to housing conditions. *Br. Poult. Sci.* **2000**, *41*, 22–28.
3. Riedstra, B.; Groothuis, T.G.G. Prenatal light exposure affects early feather-pecking behaviour in the domestic chick. *Anim. Behav.* **2004**, *67*, 1037–1042.
4. van Hierden, Y.M.; Koolhaas, J.M.; Korte, S.M. Chronic increase of dietary l-tryptophan decreases gentle feather pecking behaviour. *Appl. Anim. Behav. Sci.* **2004**, *89*, 71–84.
5. Chow, A.; Hogan, J.A. The development of feather pecking in Burmese red junglefowl: the influence of early experience with exploratory-rich environments. *Appl. Anim. Behav. Sci.* **2005**, *93*, 283–294.
6. McAdie, T.M.; Keeling, L.J.; Blokhuis, H.J.; Jones, R.B. Reduction in feather pecking and improvement of feather condition with the presentation of a string device to chickens. *Appl. Anim. Behav. Sci.* **2005**, *93*, 67–80.
7. Zimmerman, P.H.; Lindberg, A.C.; Pope, S.J.; Glen, E.; Bolhuis, J.E.; Nicol, C.J. The effect of stocking density, flock size and modified management on laying hen behaviour and welfare in a non-cage system. *Appl. Anim. Behav. Sci.* **2006**, *101*, 111–124.
8. Dixon, L.M.; Duncan, I.J.H.; Mason, G. What's in a peck? Using fixed action pattern morphology to identify the motivational basis of abnormal feather-pecking behaviour. *Anim. Behav.* **2008**, *76*, 1035–1042.
9. Lambton, S.L.; Knowles, T.G.; Yorke, C.; Nicol, C.J. The risk factors affecting the development of gentle and severe feather pecking in loose housed laying hens. *Appl. Anim. Behav. Sci.* **2010**, *123*, 32–42.
10. Collins, L.M.; Asher, L.; Pfeiffer, D.U.; Browne, W.J.; Nicol, C.J. Clustering and synchrony in laying hens: The effect of environmental resources on social dynamics. *Appl. Anim. Behav. Sci.* **2011**, *129*, 43–53.
11. Gilani, A.-M.; Knowles, T.G.; Nicol, C.J. The effect of dark brooders on feather pecking on commercial farms. *Appl. Anim. Behav. Sci.* **2012**, *142*, 42–50.
12. Hartcher, K.M.; Tran, K.T.N.; Wilkinson, S.J.; Hemsworth, P.H.; Thomson, P.C.; Cronin, G.M. The effects of environmental enrichment and beak-trimming during the rearing period on subsequent feather damage due to feather-pecking in laying hens. *Poult. Sci.* **2015**, *94*, 852–859.
13. Zepp, M.; Louton, H.; Erhard, M.; Schmidt, P.; Helmer, F.; Schwarzer, A. The influence of stocking density and enrichment on the occurrence of feather pecking and aggressive pecking behavior in laying hen chicks. *J. Vet. Behav.* **2018**, *24*, 9–18.
14. Kjaer, J.B. Diurnal rhythm of feather pecking behaviour and condition of integument in four strains of loose housed laying hens. *Appl. Anim. Behav. Sci.* **2000**, *65*, 331–347.
15. Kjaer, J.B.; Sùrensen, P. Feather pecking and cannibalism in free-range laying hens as affected by genotype, dietary level of methionine ⊚ cystine, light intensity during rearing and age at ®rst access to the range area. *Appl. Anim. Behav. Sci.* **2002**, 19.
16. Rodenburg, T.; Buitenhuis, A.; Ask, B.; Uitdehaag, K.; Koene, P.; van der Poel, J.; Bovenhuis, H. Heritability of feather pecking and open-field response of laying hens at two different ages. *Poult. Sci.* **2003**, *82*, 861–867.
17. Bolhuis, J.E.; Ellen, E.D.; Van Reenen, C.G.; De Groot, J.; Napel, J.T.; Koopmanschap, R.E.; De Vries Reilingh, G.; Uitdehaag, K.A.; Kemp, B.; Rodenburg, T.B. Effects of genetic group selection against mortality on behavior and peripheral serotonin in domestic laying hens with trimmed and intact beaks. *Physiol. Behav.* **2009**, *97*, 470–475.

18. Kjaer, J.B.; Jørgensen, H. Heart rate variability in domestic chicken lines genetically selected on feather pecking behavior. *Genes Brain Behav.* **2011**, *10*, 747–755.
19. de Haas, E.N.; Bolhuis, J.E.; Kemp, B.; Groothuis, T.G.G.; Rodenburg, T.B. Parents and Early Life Environment Affect Behavioral Development of Laying Hen Chickens. *PLoS ONE* **2014**, *9*, e90577.
20. van der Eijk, J.A.J.; Verwoolde, M.B.; de Vries Reilingh, G.; Jansen, C.A.; Rodenburg, T.B.; Lammers, A. Chicken lines divergently selected on feather pecking differ in immune characteristics. *Physiol. Behav.* **2019**, *212*, 112680.
21. Piepho, H.-P.; Lutz, V.; Kjaer, J.B.; Grashorn, M.; Bennewitz, J.; Bessei, W. The presence of extreme feather peckers in groups of laying hens. *animal* **2017**, *11*, 500–506.
22. Iffland, H.; Wellmann, R.; Preuß, S.; Tetens, J.; Bessei, W.; Piepho, H.-P.; Bennewitz, J. A Novel Model to Explain Extreme Feather Pecking Behavior in Laying Hens. *Behav. Genet.* **2019**.
23. Albentosa, M.J.; Glen, E.; Leeb, C.; Whittaker, X.; Nicol, C.J. An evaluation of response to novelty as a predictor of pecking tendency in laying hens. *Appl. Anim. Behav. Sci.* **2003**, *82*, 313–328.
24. de Haas, E.N.; Kemp, B.; Bolhuis, J.E.; Groothuis, T.; Rodenburg, T.B. Fear, stress, and feather pecking in commercial white and brown laying hen parent-stock flocks and their relationships with production parameters. *Poult. Sci.* **2013**, *92*, 2259–2269.
25. van Hierden, Y.M.; de Boer, S.F.; Koolhaas, J.M.; Korte, S.M. The Control of Feather Pecking by Serotonin. *Behav. Neurosci.* **2004**, *118*, 575–583.
26. Parmentier, H.K.; Rodenburg, T.B.; De Vries Reilingh, G.; Beerda, B.; Kemp, B. Does enhancement of specific immune responses predispose laying hens for feather pecking? *Poult. Sci.* **2009**, *88*, 536–542.
27. Kops, M.S.; de Haas, E.N.; Rodenburg, T.B.; Ellen, E.D.; Korte-Bouws, G.A.H.; Olivier, B.; Güntürkün, O.; Bolhuis, J.E.; Korte, S.M. Effects of feather pecking phenotype (severe feather peckers, victims and non-peckers) on serotonergic and dopaminergic activity in four brain areas of laying hens (Gallus gallus domesticus). *Physiol. Behav.* **2013**, *120*, 77–82.
28. van Hierden, Y.M.; Korte, S.M.; Ruesink, E.W.; van Reenen, C.G.; Engel, B.; Koolhaas, J.M.; Blokhuis, H.J. The development of feather pecking behaviour and targeting of pecking in chicks from a high and low feather pecking line of laying hens. *Appl. Anim. Behav. Sci.* **2002**, *77*, 183–196.
29. Cloutier, S.; Newberry, R.C.; Honda, K.; Alldredge, J.R. Cannibalistic behaviour spread by social learning. *Anim. Behav.* **2002**, *63*, 1153–1162.
30. Forkman, B.; Heiskanen, T.; Kjær, J. 2004 SPRING MEETING OF THE WPSA UK BRANCH PAPERS: The predictions from coping theory are not upheld for severe featherpeckers. *Br. Poult. Sci.* **2004**, *45*, S34–S35.
31. Harlander-Matauschek, A.; Benda, I.; Lavetti, C.; Djukic, M.; Bessei, W. The relative preferences for wood shavings or feathers in high and low feather pecking birds. *Appl. Anim. Behav. Sci.* **2007**, *107*, 78–87.

© 2020 by the authors. Submitted for possible open access publication under the terms and conditions of the Creative Commons Attribution (CC BY) license (http://creativecommons.org/licenses/by/4.0/).
